# Genetic and Molecular Insights into Transforming Growth Factor-Beta Signaling in Periodontitis: A Systematic Review

**DOI:** 10.3390/genes16101165

**Published:** 2025-10-01

**Authors:** Tomasz Pawłaszek, Beniamin Oskar Grabarek

**Affiliations:** 1 Collegium Medicum, WSB University, 41-300 Dąbrowa Górnicza, Poland; bgrabarek7@gmail.com; 2 PROTOM Dental, 32-500 Chrzanów, Poland

**Keywords:** transforming growth factor-beta, TGF-β1, periodontitis, gingival crevicular fluid, cytokines, biomarkers, inflammation, regenerative therapy, SMAD signaling, periodontal disease

## Abstract

**Background/Objectives:** Transforming growth factor-beta (TGF-β) is a multifunctional cytokine involved in immune regulation, extracellular matrix turnover, and tissue repair. Its role in periodontitis remains controversial due to conflicting human studies. This systematic review addressed the PICO-based question: in adults with periodontitis (population), how does the expression and regulation of TGF-β isoforms (intervention/exposure) compare with healthy or post-treatment states (comparator) regarding clinical outcomes (outcomes)? **Methods:** A systematic search of PubMed and Scopus was conducted on 1 July 2025 for human studies published in English between 2010 and 2025. Eligible studies investigated TGF-β expression, function, or genetic regulation in periodontal tissues or biological fluids. Screening and quality appraisal were performed according to PRISMA guidelines, using design-specific risk-of-bias tools. The review protocol was prospectively registered in PROSPERO (CRD420251138456). **Results:** Fifteen studies met inclusion criteria. TGF-β1 was the most frequently analyzed isoform and was consistently elevated in diseased gingival tissue and gingival crevicular fluid, correlating with probing depth and attachment loss. Several studies reported post-treatment reductions in TGF-β, supporting its value as a dynamic biomarker. Additional findings linked TGF-β signaling to immune modulation, fibrosis, bone turnover, and systemic comorbidities. Evidence for TGF-β2 and TGF-β3 was limited but suggested isoform-specific roles in epithelial–mesenchymal signaling and scar-free repair. **Conclusions:** Current evidence supports TGF-β, particularly TGF-β1, as a central mediator of periodontal inflammation and repair, with promise as both a biomarker and therapeutic target. Standardized, isoform-specific, and longitudinal studies are needed to clarify its diagnostic and translational utility.

## 1. Introduction

Periodontitis is one of the most prevalent chronic inflammatory diseases worldwide and remains a major cause of tooth loss in adults. It is now understood not merely as a localized infection but as a complex, multifactorial condition initiated by dysbiotic biofilms and sustained by a dysregulated host immune–inflammatory response. This interplay leads to progressive destruction of the periodontal ligament and alveolar bone, ultimately compromising tooth support and oral function [1,2]. Beyond the oral cavity, periodontitis has been consistently associated with systemic disorders such as diabetes mellitus, cardiovascular disease, and chronic kidney disease, underscoring its relevance to general health [3,4,5].

To improve diagnostic precision and international comparability, the 2017 World Workshop on the Classification of Periodontal and Peri-Implant Diseases and Conditions introduced a staging and grading framework. Staging reflects the severity and extent of periodontal destruction, ranging from stage I (incipient disease) to stage IV (advanced disease with functional impairment), while grading estimates the rate of progression and risk factors, with grade A indicating slow progression, grade B moderate progression, and grade C rapid progression. This updated classification has become the global standard and emphasizes the need to integrate clinical, biological, and risk-related factors when evaluating periodontitis [6].

Within this framework, attention has increasingly turned to molecular mediators such as transforming growth factor-beta (TGF-β), a pleiotropic cytokine [7]. In mammals, three isoforms—TGF-β1, TGF-β2, and TGF-β3—are expressed, with TGF-β1 being the most abundantly produced and studied in periodontal tissue [8]. The biological actions of TGF-β are mediated via a serine/threonine kinase receptor complex and subsequent activation of intracellular signaling cascades, primarily through the Mothers Against Decapentaplegic Homolog (SMAD)-dependent canonical pathway and alternative non-canonical pathways (e.g., mitogen-activated protein kinase (MAPK), phosphoinositide 3-kinase/protein kinase B (PI3K/AKT)). These pathways culminate in the regulation of gene expression involved in inflammation, extracellular matrix (ECM) remodeling, and cell differentiation [9,10,11].

In the context of periodontitis, TGF-β plays a paradoxical role [12]. On one hand, it promotes the resolution of inflammation, supports the survival of periodontal ligament fibroblasts, and stimulates collagen synthesis and bone matrix deposition, thereby contributing to tissue repair and regeneration [13,14]. On the other hand, dysregulated or chronic activation of TGF-β signaling has been implicated in pathological fibrosis, aberrant immune responses, and osteoclastogenesis, which exacerbate periodontal destruction [15,16]. Moreover, recent studies suggest that TGF-β interacts with other key mediators such as tumor necrosis factor-alpha (TNF-α), interleukin-1β (IL-1β), and receptor activator of nuclear factor κB ligand (RANKL), forming complex regulatory networks that influence disease outcome [17].

Given the growing interest in TGF-β as a potential therapeutic target, especially in regenerative periodontal therapy and biomaterial engineering, it is crucial to comprehensively understand its role in the pathogenesis and progression of periodontitis [18,19,20].

Despite the substantial body of literature, the findings regarding TGF-β in periodontitis remain heterogeneous and occasionally contradictory, owing to differences in study designs, patient populations, sampling techniques, and analytical methods. To date, no systematic review has comprehensively synthesized the current evidence on the role of TGF-β in periodontitis across molecular, cellular, and clinical levels.

Therefore, the objective of this systematic review was to critically evaluate and summarize current evidence on the role of TGF-β in periodontitis by explicitly addressing a PICO-based research question: in adult patients with periodontitis, what is the role of TGF-β isoforms compared with healthy or post-treatment conditions in relation to clinical severity, treatment outcomes, and systemic associations? In pursuing this aim, this review sought to integrate findings on differential isoform expression in human periodontal tissues and fluids to explore the regulatory mechanisms underlying TGF-β signaling at the molecular and genetic level and to determine the extent to which these pathways influence disease activity, response to therapy, and potential diagnostic or therapeutic applications.

## 2. Materials and Methods

### 2.1. Search Strategy

A systematic literature search was independently performed by two investigators (T.P. and B.O.G.) in PubMed and Scopus on 1 July 2025. The combined search yielded 11,601 records, of which 329 were from PubMed and 11,272 from Scopus. Search terms included multiple variants of transforming growth factor-beta combined with periodontal disease descriptors, for example, “transforming growth factor-beta,” “TGF-beta,” “TGF-β,” and “periodontitis” or “periodontal disease.” Initial filters were applied to restrict the dataset to articles published from 2010 onwards, original research studies, and adult human populations. The review protocol was prospectively registered in the International Prospective Register of Systematic Reviews (PROSPERO; registration ID CRD420251138456).

### 2.2. Eligibility Criteria and Study Selection

We included original, peer-reviewed human studies published in English between 2010 and 2025 that investigated TGF-β in the context of periodontal disease. Eligible designs comprised randomized controlled trials (RCTs), cohort, case–control, cross-sectional, genetic association, and controlled pre–post studies. Adult participants (≥18 years) with periodontal conditions (e.g., gingivitis, chronic/aggressive periodontitis, chronic apical periodontitis) were eligible. Biological matrices included gingival tissue, gingival crevicular fluid (GCF), saliva, serum, alveolar bone, and periapical lesion tissues. Key outcomes were TGF-β isoform expression (protein or mRNA), associations with clinical indices (e.g., PI, GI, PPD, CAL), and pre–post therapeutic change. Exclusion criteria were non-human studies, in vitro-only work without human validation, reviews, editorials, conference abstracts, and non-English reports. We restricted inclusion to studies with free full-text availability to ensure full methodological transparency and reproducibility of data extraction and quality appraisal; this allowed verification of sampling, laboratory procedures, and statistical analyses beyond abstracts. The final dataset remained diverse in geography and design despite this restriction.

Two reviewers (T.P. and B.O.G.) independently screened titles, abstracts, and full texts for eligibility. Inter-rater agreement was quantified using Cohen’s kappa (κ = 0.82), indicating very good concordance. Any disagreements were subsequently resolved through discussion until consensus was reached.

### 2.3. Risk of Bias Assessment

Study quality was evaluated with validated, design-specific tools. Randomized controlled trials were appraised with the Cochrane risk of bias 2 (RoB 2) instrument [21], which evaluates five domains: randomization process, adherence to interventions, missing outcome data, measurement of outcomes, and selective reporting. Observational and non-randomized studies, including cohort, case–control, cross-sectional, genetic association, and controlled pre–post designs, were assessed with a modified Newcastle–Ottawa scale (NOS), which considers selection, comparability, and outcome or exposure, with scores ranging from 0 to 9. For interpretability, scores were categorized as good (7–9), moderate (5–6), or poor (≤4) [22].

Study quality was independently evaluated by the same two reviewers using design-specific tools. Agreement on risk of bias ratings was assessed with Cohen’s kappa (κ = 0.82), confirming very good inter-rater reliability. Any discrepancies in scoring were discussed until consensus was achieved.

### 2.4. Data Extraction and Synthesis (Added)

Because of considerable methodological heterogeneity across studies—including differences in study design, biological matrices (gingival tissue, GCF, serum, saliva, periapical tissue), analytical techniques (RT-qPCR, ELISA, immunohistochemistry), and reporting formats (absolute concentrations, relative expression, or categorical outcomes)—a formal meta-analysis was not feasible. In particular, inconsistent units of measurement, lack of standardized cut-off values, and variable post-treatment follow-up times precluded quantitative pooling. For these reasons, a narrative synthesis was performed. However, wherever possible, quantitative results were summarized descriptively to highlight patterns in biomarker expression and treatment-related changes. Data extraction was conducted independently by two reviewers (T.P. and B.O.G.) using a standardized extraction form piloted on three studies. Extracted variables included study design, population, biological sample, TGF-β isoform(s) analyzed, methods of detection, and main outcomes. Any discrepancies were resolved through consensus. No automation tools were used for data collection, “No formal assessment of reporting bias (e.g., funnel plots or Egger’s test) or certainty of evidence grading (e.g., GRADE) was conducted due to the small number and heterogeneity of included studies. This is acknowledged as a limitation.

## 3. Results

### 3.1. Study Selection

**Figure 1:**
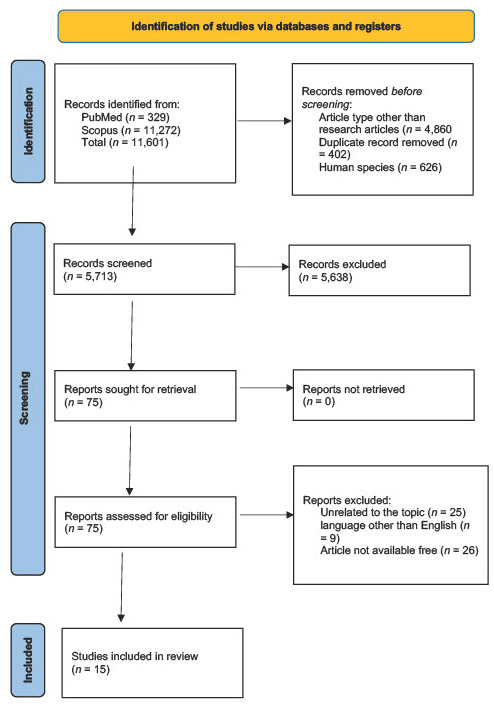
PRISMA flow diagram of systematic review [23].

The initial database search identified 11,601 records related to TGF-β and periodontitis, with 329 retrieved from PubMed and 11,272 from Scopus. After removal of duplicates and application of eligibility filters—including publication year (2010 or later), study type (original research), and human adult populations—the pool of records was substantially reduced. In PubMed, 21 articles satisfied the inclusion requirements of being in English, freely accessible in full text, and conducted in adult participants. From Scopus, an initial set of more than ten thousand articles was progressively narrowed by full-text availability, language, and population criteria, leaving 575 studies for detailed review. Following full-text screening across both databases, 23 articles appeared eligible. After deduplication and exclusion of non-original designs, in vitro-only work, and animal studies, a total of 15 original human studies were retained for qualitative synthesis. These included two randomized controlled trials and thirteen observational or non-randomized studies. The selection process is summarized in the preferred reporting items for systematic reviews and meta-analyses (PRISMA) flow diagram (Figure 1).

#### Risk of Bias (Updated)

Among the two randomized controlled trials, one was judged to have overall low risk of bias, while the other was rated as having some concerns because of unblinded surgical interventions and unclear reporting due to absent preregistration. The thirteen observational or non-randomized studies achieved a median NOS score of 6 out of 9 (interquartile range 6–7), which indicates moderate to good quality. The most common limitations were small sample sizes, convenience sampling, and incomplete adjustment for potential confounders such as age, smoking, or comorbidities. In contrast, strengths included clear case definitions of periodontal disease and the use of standardized laboratory assays, particularly RT-qPCR, ELISA, and immunohistochemistry. Detailed study-level ratings are presented in Table 1 for randomized controlled trials and in Table 2 for observational studies.

### 3.2. Characteristics of the Included Studies

The included studies were published between 2010 and 2025 and originated from multiple geographic regions, with Brazil contributing six studies, followed by the United States, Japan, China, and several European and Asian countries. Most investigations used cross-sectional designs, although interventional protocols and randomized controlled trials were also represented. A single study used a genetic association design to examine polymorphisms in the TGFB3 gene.

Study populations consisted of adults with chronic or aggressive periodontitis, gingivitis, or chronic apical periodontitis. Some studies also investigated patients with systemic comorbidities such as chronic kidney disease, reflecting the broader systemic associations of periodontal inflammation. To enhance internal validity, several investigations included comparison groups such as healthy controls, post-treatment patients, or individuals with clinically stable periodontal conditions.

The biological materials examined varied across studies. Gingival tissue and periodontal ligament were the most frequently analyzed, followed by gingival crevicular fluid, serum, saliva, and periapical lesion tissue. Nine studies explicitly quantified TGF-β1 expression at either the protein or mRNA level, while others reported findings for total TGF-β or examined downstream mediators. Only one study specifically addressed TGFB3 polymorphisms and their potential link to disease susceptibility (Table 3).

## 4. Discussion

This systematic review synthesized evidence from original human studies investigating the involvement of transforming growth factor-beta (TGF-β) in the pathophysiology of periodontitis. Collectively, the findings highlight TGF-β—particularly TGF-β1—as a pivotal regulator of periodontal inflammation, extracellular matrix (ECM) remodeling, and wound-healing processes [39,40]. Elevated levels of TGF-β1 were consistently observed in gingival tissues and gingival crevicular fluid (GCF) of patients with chronic periodontitis, where they correlated with established clinical indices such as probing pocket depth (PPD), clinical attachment level (CAL), and plaque index (PI) [28,35]. Importantly, several studies demonstrated that TGF-β concentrations decrease after non-surgical periodontal treatment (NSPT) or surgical interventions, underscoring TGF-β’s potential role as a dynamic biomarker of therapeutic response [24,32,36].

From a clinical perspective, TGF-β can be viewed as a mediator at the intersection of destructive inflammation and regenerative repair. Escobar et al. [32] and Dengizek et al. [24] reported that periodontal therapy not only improves clinical parameters but also significantly alters TGF-β expression profiles. Similarly, Sattari et al. [36] observed postoperative reductions in GCF TGF-β1, consistent with the resolution of active inflammation. These findings suggest that monitoring TGF-β1 dynamics could aid clinicians in evaluating treatment efficacy and tailoring retreatment strategies. Beyond biomarker applications, regenerative studies provide evidence that TGF-β may actively contribute to repair. Liang et al. [25], for instance, demonstrated that guided tissue regeneration combined with anchorage techniques enhanced TGF-β expression in GCF while reducing matrix-degrading factors, thereby promoting a regenerative environment. Such observations highlight the therapeutic potential of isoform-targeted approaches—for example, leveraging TGF-β3’s antifibrotic and pro-regenerative properties in periodontal tissue engineering.

Beyond local tissue effects, several studies emphasized the systemic relevance of TGF-β in patients with comorbid conditions. Rajaratinam et al. [30] demonstrated that NSPT significantly reduced serum TGF-β1 and IL-6 in patients with chronic kidney disease, accompanied by improved renal function (eGFR). This underscores the potential bidirectional relationship between periodontal therapy, systemic inflammation, and organ health. In another context, Panezai et al. [28] found correlations between serum TGF-β1 levels and periodontal status in rheumatoid arthritis patients, suggesting that systemic monitoring of TGF-β may have diagnostic or prognostic value across different inflammatory diseases. The functional relevance of TGF-β extends beyond biomarker dynamics. Toledo et al. [37] and Popović et al. [29] highlighted its role in shaping the Th17/Treg balance and in stabilizing chronic inflammatory lesions, consistent with the broader role of TGF-β in immune homeostasis. Da Costa et al. [38] and Cirano et al. [26] further linked TGF-β expression to tissue fibrosis and altered bone turnover in aggressive forms of periodontitis, suggesting that dysregulated signaling can promote pathological remodeling. Importantly, genetic variation may influence individual susceptibility. Gonçalves Junior et al. [33] investigated TGF-β3 polymorphisms in Brazilian cohorts, showing that chronic periodontitis significantly increased peri-implantitis risk, although no direct association with TGFB3 variants was confirmed. These results argue for further isoform-specific genetic and epigenetic studies to elucidate host susceptibility patterns.

Although TGF-β1 dominated the included literature, isoform-specific biology suggests complementary roles for TGF-β2 and TGF-β3 in periodontal homeostasis and repair [41]. TGF-β2 has been implicated in epithelial–mesenchymal crosstalk, epithelial barrier integrity, and osteo-immune coupling, indicating a potential role at the interface of host defense and bone remodeling [42,43]. In contrast, TGF-β3 is repeatedly linked to scar-modulating, pro-regenerative signaling, and antifibrotic wound healing phenotypes that differ from TGF-β1 [44,45,46]. Within our corpus, isoform-resolved clinical data for TGF-β2/β3 remain sparse; notably, one genetic association study did not find a significant relationship between a TGF-β-3 polymorphism and periodontitis or peri-implantitis, underscoring that isoform-specific effects may be context-dependent and subtle at the population level. Taken together, these observations argue for future isoform-resolved studies combining quantitative assays (active vs. latent protein forms), spatial profiling in gingival and bone compartments, and functional readouts (e.g., ECM turnover, osteoclastogenesis, Treg/Th17 balance) [33]. Such designs are essential to determine whether TGF-β-2 signals align more with mucosal protection and whether TGF-β-3 could be harnessed to bias healing toward antifibrotic, regenerative outcomes in periodontitis.

From a biomarker perspective, TGF-β—particularly TGF-β-1—behaves as a dynamic marker of disease activity and treatment response across matrices (GCF, saliva, serum, tissue). Before clinical adoption, three prerequisites are needed: (i) assay standardization (active vs. latent forms, kit/vendor, calibration, pre-analytics, normalization), (ii) matrix-specific reference ranges and analytical cut-offs established in multicenter cohorts, and (iii) evaluation within multimarker panels (e.g., TGF-β with IL-6, MMP-8, RANKL/OPG) to improve discrimination beyond single-analyte performance. Practical use cases include risk stratification at baseline, monitoring response after non-surgical therapy or surgery, and supporting decisions for retreatment [47,48].

Therapeutically, global TGF-β inhibition may carry off-target risks given its central homeostatic roles. More plausible strategies for periodontology are context- and isoform-targeted: (i) local delivery (e.g., GTR membranes, hydrogels, microspheres) to confine exposure; (ii) pro-regenerative biasing with TGF-β-3 to reduce fibrosis and improve matrix architecture in intrabony defects; and (iii) pathway-selective modulation (e.g., canonical vs. non-canonical SMAD/MAPK nodes) to uncouple repair from pro-fibrotic signaling. Early-phase trials should incorporate pharmacodynamic biomarkers (local TGF-β activity, ECM turnover markers) and safety readouts to define therapeutic windows [49,50,51,52].

In summary, the reviewed literature consistently supports the central role of TGF-β—predominantly TGF-β1—as a multifaceted mediator of periodontal disease progression and resolution [53,54]. Its expression levels were found to correlate with clinical severity, treatment response, systemic inflammatory burden, and, in some cases, genetic predisposition. These data reinforce the utility of TGF-β as both a biomarker of disease activity and a potential therapeutic target for modulating host responses in periodontitis [20,55].

We acknowledge that the restriction to PubMed and Scopus, as well as to English-language and freely accessible full-text publications, may introduce both language and publication bias and limit the comprehensiveness of the review. Important studies indexed in Embase, Web of Science, or the Cochrane Library and non-English articles could therefore have been overlooked. Expanding future searches to include additional databases and incorporating studies in other languages with appropriate translation support would improve the breadth and representativeness of the evidence base.

Beyond these search-related issues, several additional limitations must be considered. Considerable heterogeneity in study designs, population characteristics, and detection methods (e.g., RT-qPCR, ELISA, immunohistochemistry) complicates direct comparisons. Many investigations relied on systemic samples such as serum, which may not fully reflect local tissue-level activity, while the lack of standardized diagnostic criteria for periodontal disease further weakened cross-study comparability. The overwhelming focus on TGF-β1 has left the roles of TGF-β2 and TGF-β3 largely underexplored, despite their potential contributions to periodontal biology. Moreover, most studies were cross-sectional or short-term, limiting insight into longitudinal changes and long-term treatment outcomes. Although several included studies provided quantitative biomarker data, methodological heterogeneity in assay types, reporting standards, and clinical outcome measures prevented meaningful statistical pooling. Consequently, only a narrative synthesis was conducted. We did not formally assess reporting biases or certainty of evidence (GRADE). Therefore, the overall strength of the evidence should be interpreted cautiously, particularly given the modest number of included studies and their methodological heterogeneity.

Future research should therefore integrate both experimental and clinical approaches. Experimental directions include isoform-specific profiling of TGF-β activity (distinguishing active vs. latent forms), multi-omics approaches to map downstream signaling networks (SMAD vs. MAPK/PI3K/AKT pathways), and the development of biomaterials for localized, controlled release of TGF-β modulators. Clinically, longitudinal studies should evaluate TGF-β as a chairside biomarker in saliva or GCF, with standardized cut-offs and assay harmonization. TGF-β should also be tested within multimarker diagnostic panels alongside IL-6, MMP-8, or RANKL/OPG to enhance predictive accuracy. Finally, translational trials of isoform-targeted strategies—such as TGF-β3 supplementation to reduce fibrosis or selective SMAD/MAPK modulation—may provide novel therapeutic avenues in periodontology. Importantly, such approaches should include safety assessments, given the pleiotropic and systemic functions of TGF-β.

## 5. Conclusions

The current evidence demonstrates that TGF-β, particularly TGF-β1, is consistently elevated in periodontal disease and correlates with clinical indices of severity. Its levels respond dynamically to therapy, underscoring its potential value as a biomarker for disease monitoring and treatment evaluation. At the same time, functional studies indicate that TGF-β is not merely a marker but an active regulator of immune balance, extracellular matrix remodeling, and tissue repair within the periodontal microenvironment. Importantly, the dual nature of TGF-β—as both a pro-regenerative and pro-fibrotic factor—suggests that its therapeutic targeting must be carefully context-specific. Isoform-selective approaches, especially those harnessing the antifibrotic and regenerative potential of TGF-β3, may offer novel strategies to enhance periodontal regeneration while minimizing pathological fibrosis. However, before clinical translation can be realized, several challenges must be addressed: assay standardization, establishment of reference ranges in saliva and GCF, integration into multimarker diagnostic panels, and validation in longitudinal and multicenter cohorts. Additionally, the roles of TGF-β2 and TGF-β3, as well as genetic and epigenetic modifiers of TGF-β signaling, remain underexplored. In conclusion, TGF-β represents both a promising biomarker and a potential therapeutic target in periodontitis. Its incorporation into future diagnostic tools and regenerative therapies will depend on isoform-specific investigations, translational trials, and rigorous methodological harmonization. By bridging molecular insights with clinical application, TGF-β may ultimately contribute to more precise diagnosis, improved monitoring, and innovative treatment strategies in periodontology.

## Figures and Tables

**Table 1:** Cochrane Risk of Bias 2 (RoB 2) assessment of randomized controlled trials.

Study (Year)	Design	D1 Randomization	D2 Deviations	D3 Missing Data	D4 Outcome Measurement	D5 Reported Result	Overall RoB 2
Dengizek et al. (2019) [24]	Randomized, double-blind, placebo-controlled RCT	Low	Low	Low	Low	Low	Low risk
Liang et al. (2025) [25]	Randomized parallel-group surgical RCT	Low	Some concerns	Low	Low	Some concerns	Some concerns

RCT, randomized controlled trial; RoB 2, risk of bias 2; D1, randomization process; D2, deviations from intended interventions; D3, missing outcome data; D4, outcome measurement; D5, selection of the reported result.

**Table 2:** NOS assessment of observational and non-randomized studies.

Study (Year)	Design	Biological Matrix	TGF-β Isoform	NOS (0–9)	NOS Category	Key Limitations/Notes
Cirano et al. (2020) [26]	Cross-sectional (GAP on SPT vs. healthy)	Alveolar bone biopsies (qRT-PCR)	TGF-β (mRNA)	7	good	Examiner-blind; rigorous inclusion/exclusion; smokers excluded; matched demographics; no multivariable models
Lorenzi et al. (2014) [27]	Cross-sectional (healthy/gingivitis/CP/AgP)	Gingival tissue (IHC + RT-PCR)	Indirect (HtrA1 inhibits TGF-β)	7	good	Rigorous inclusion/exclusion; clear phenotypes; indirect readout; no multivariable adjustment
Panezai et al. (2017) [28]	Cross-sectional (RA, PD, healthy)	Serum (Olink PEA 92-plex)	LAP TGF-β1 (latent complex)	7	good	Standardized acquisition; multiplicity control (FDR); convenience sampling; limited confounder adjustment
Popović et al. (2025) [29]	Cross-sectional (symptomatic vs. asymptomatic lesions)	Periapical lesion tissue (ELISA + PCR for viruses)	TGF-β1	7	good	Clear case definition; blinded lab assays implied; limited adjustment for confounders
Rajaratinam et al. (2025) [30]	Prospective controlled (CKD-P vs. P vs. healthy)	Serum (TGF-β1, IL-6) + clinical + eGFR	TGF-β1	7	good	Prospective with pre/post; nonrandomized; standardized measures; confounding by indication possible
Dessaune Neto et al. (2018) [31]	Cross-sectional (post-treatment apical periodontitis)	Periapical lesion tissue (IHC)	TGF-β (unspecified)	6	moderate	Standardized IHC scoring; two blinded evaluators; convenience surgical sample; limited confounder control
Escobar et al. (2018) [32]	Controlled before–after (NSPT) + healthy controls	Serum (cytokines/CRP)	TGF-β (reported)	6	moderate	Pre/post within CP; nonrandomized; potential selection bias; standard assays
Gonçalves Junior et al. (2016) [33]	Cross-sectional genetic association (CP and/or PI)	Genotyping (buccal epithelial cells)	TGF-β3 polymorphism (rs2268626)	6	moderate	Well-defined groups; blinded lab workflow; no multivariable adjustment; cross-sectional design
Honda et al. (2006) [34]	Case–control (gingivitis vs. periodontitis)	Gingival biopsies (qRT-PCR)	TGF-β1 mRNA	6	moderate	Clear case definitions; age imbalance; no adjustment for confounding; blinded lab methods not stated
Mize et al. (2015) [35]	Case–control (periodontitis vs. non-periodontitis)	Gingival tissue (qRT-PCR)	TGF-β1 mRNA	6	moderate	Clear criteria; small sample; non-matched controls; no multivariable adjustment
Sattari et al. (2011) [36]	Case–control (PD vs healthy); IHC	Alveolar bone/tissues (IHC)	TGF-β (unspecified)	6	moderate	Convenience surgical samples; no matching; standard IHC; potential age/smoking imbalance
Toledo et al. (2019) [37]	Cross-sectional (periapical lesions vs. necrotic pulp vs. control)	Periapical tissue homogenates (ELISA)	TGF-β (unspecified)	6	moderate	Clear group definitions; histopathology confirmation; standardized ELISA; limited confounder control
da Costa et al. (2015) [38]	Case–control (advanced CP vs. controls)	Alveolar bone and adjacent tissues (IHC for TGF-β/IL-17; qRT-PCR)	TGF-β (protein by IHC)	6	moderate	Clear criteria; surgical bone biopsies; smokers present; age imbalance; no multivariable adjustment; standardized IHC/qPCR

AgP, aggressive periodontitis; CKD-P, chronic kidney disease with periodontitis; CP, chronic periodontitis; CRP, C-reactive protein; eGFR, estimated glomerular filtration rate; ELISA, enzyme-linked immunosorbent assay; FDR, false discovery rate; GAP, generalized aggressive periodontitis; HtrA1, high temperature requirement A serine peptidase 1; IHC, immunohistochemistry; IL-6, interleukin-6; LAP, latency-associated peptide; NOS, Newcastle–Ottawa Scale; NSPT, non-surgical periodontal treatment; PD, periodontal disease; PEA, proximity extension assay; PI, peri-implantitis; qRT-PCR, quantitative real-time polymerase chain reaction; RA, rheumatoid arthritis; TGF-β, transforming growth factor-beta.

**Table 3:** Characteristics of the included studies.

First Author (Year)	Country	Population	Biological Material	TGF-β Isoform(s)	Main Outcomes	Key Findings
Mize et al. (2015) [35]	USA	21 individuals (7 healthy, 14 with chronic periodontitis)	Gingival tissue	TGF-β1	To assess expression of TGF-β1 and CTGF/CCN2 in gingiva of patients with periodontitis	TGF-β1 and CTGF/CCN2 mRNA expression levels were significantly increased and positively correlated in periodontitis tissues.
Panezai et al. (2017) [28]	Sweden	90 individuals (38 with RA, 38 with PD, 14 healthy controls)	Serum	TGF-β1 (LAP TGF-β1)	To investigate serum cytokines, chemokines, growth factors, and enzymes in relation to periodontal disease parameters	LAP TGF-β1 was positively associated with the number of teeth and inversely associated with shallow pockets; systemic inflammatory markers including TGF-β1 correlate with periodontal status in PD and RA patients.
Gonçalves Junior et al. (2016) [33]	Brazil	163 individuals with or without history of chronic periodontitis and/or peri-implantitis	Buccal epithelial cells (for genotyping)	TGF-β3	To evaluate the relationship between CP, PI, and gene polymorphisms in MMP13, TIMP2, and TGFB3	CP significantly increased risk of PI (OR = 3.2), but no significant association was found between TGFB3 (rs2268626) polymorphism and CP or PI.
Dessaune Neto et al. (2018) [31]	Brazil	27 patients with apical periodontitis undergoing periradicular surgery	Apical lesion tissue (granulomas and cysts)	TGF-β (unspecified isoform) GF-β	To compare pro- and anti-inflammatory cytokine expression and correlate with clinical/CBCT data	TGF-β expression was weak/moderate most often, significantly associated with cyst lesions treated ≤4 years earlier (*p* = 0.045); overall cytokine expression showed balance between pro- and anti-inflammatory mediators.
Cirano et al. (2020) [26]	Brazil	33 young adults (16 with generalized aggressive periodontitis (GAP), 17 healthy controls)	Alveolar bone biopsies	TGF-β (unspecified)	To compare gene expression of bone turnover markers including TGF-β in alveolar bone between GAP and healthy individuals	Patients with GAP exhibited significantly lower TGF-β and OPG mRNA levels compared to healthy controls (*p* ≤ 0.05); no differences in TNF-α, RANKL, OC, BSP, COL-I expression.
Toledo et al. (2019) [37]	Brazil	86 human teeth: necrotic pulp with lesion (*n* = 26), necrotic pulp without lesion (*n* = 30), healthy (*n* = 30)	Periapical tissue homogenates (ELISA)	TGF-β (isoform not specified)	To investigate the role of Treg (TGF-β, IL-10, CCL4) and Th17 (IL-17, CCL20) cytokines in periapical lesions	I TGF-β and CCL4 were significantly elevated in periapical lesions vs. necrotic pulp without lesion. Weak positive correlation observed between IL-17/TGF-β, indicating co-stimulation. TGF-β may help stabilize chronic periapical inflammation.
da Costa et al. (2015) [38]	Brazil	Chronic periodontitis patients (*n* not stated)	Non-gingival affected tissues	TGF-β (unspecified)	To identify inflammatory biomarkers in tissues affected by chronic periodontitis	elevated levels of TGF-β observed in advanced non-gingival affected tissues of chronic periodontitis patients, suggesting involvement in local inflammatory response and tissue remodeling.
Sattari et al. (2011) [36]	Iran	10 patients with moderate to severe chronic periodontitis undergoing flap surgery	Gingival crevicular fluid (GCF	TGF-β1	To evaluate the effect of flap surgery on IL-1β and TGF-β1 concentrations in GCF	GF-β1 and IL-1β levels significantly decreased from baseline to 12 weeks post-surgery (*p* < 0.05); TGF-β1 levels correlated with plaque index and PPD; reduction attributed to resolution of inflammation.
Escobar et al. (2018) [32]	Brazil	40 individuals: 20 healthy patients, 20 with chronic periodontitis	Gingival crevicular fluid (GCF), serum	TGF-β (unspecified)	To compare IFN-γ, TGF-β and CRP levels in HP vs. CP before and after non-surgical periodontal treatment (NSPT)	TGF-β levels in GCF were significantly higher in CP before treatment than in HP; TGF-β levels decreased post-NSPT; early immune response was marked by decreased TGF-β and increased IFN-γ.
Dengizek et al. (2019) [24]	Turkey	40 patients with chronic periodontitis (20 SRP + ozone, 20 SRP + placebo)	Whole saliva (pre- and 1 month post-treatment)	TGF-β (unspecified)	To evaluate effects of SRP + ozone vs. SRP + placebo on clinical parameters and salivary biochemical markers	TGF-β levels significantly increased only in the ozone group post-treatment (*p* < 0.05), suggesting enhanced tissue regenerative response. However, clinical recovery parameters (PI, GI, PD, CAL) showed no significant intergroup difference.
Lorenzi et al. (2014) [27]	Italy	56 subjects (healthy, gingivitis, CP, AgP)	Gingival biopsies	Implicit (via HtrA1)	Expression of HtrA1 and its role in inflammation and matrix degradation	HtrA1 inhibits TGF-β, promoting MMPs, IL-1β, and TNF-α; strongest expression seen in aggressive periodontitis.
Popović et al. (2025) [29]	Serbia	79 patients with chronic apical periodontitis (43 symptomatic, 36 asymptomatic)	Periapical lesion tissue	TGF-β1	Evaluate the presence of HCMV and EBV in symptomatic vs. asymptomatic lesions and assess IL-6, IL-8, TNF-α, and TGF-β1 levels via ELISA	HCMV and dual HCMV/EBV infection were significantly more frequent in symptomatic lesions. TGF-β1 was higher in virus-positive vs. virus-negative lesions but significantly so only in dual HCMV/EBV infections. IL-6, IL-8, and TNF-α were elevated in all virus-positive lesions, especially in dual infections. TGF-β1’s role may be limited in this setting.
Rajaratinam et al. (2025) [30]	Malaysia	CKD-stage 3–4 with periodontitis (n = 20), periodontitis only (n = 20), healthy (n = 20)	Serum	TGF-β1	Effects of NSPT on periodontal health, renal function (eGFR), IL-6, and TGF-β1 levels	CKD-P group had highest baseline TGF-β1. After NSPT, TGF-β1 significantly decreased only in CKD-P group (*p* < 0.001). IL-6 dropped in both CKD-P and P groups; eGFR improved in CKD-P (*p* < 0.0001).
Honda et al. (2006) [34]	Japan	Patients with stable gingivitis and progressive periodontitis	Gingival tissue/fluid	TGF-β1	Compare cytokine balance—including TGF-β—in stable vs. progressive periodontal lesions	TGF-β1 is part of the regulatory cytokine network governing stability; progressive lesions show altered pro-/anti-inflammatory balance with dysregulated TGF-β expression.
Liang et al. (2025) [25]	China	Patients undergoing GTR + microscrew anchorage for periodontitis with malocclusion	Gingival crevicular fluid (GCF)	TGF-β (presumably TGF-β1)	Effects of GTR + anchorage on GCF biomarkers including IL-6, MMP-8, and TGF-β post-treatment	Six weeks post-treatment, IL-6 and MMP-8 levels decreased significantly, while TGF-β levels increased compared to controls.

TGF-β, transforming growth factor-beta; TGF-β1, transforming growth factor-beta 1; TGF-β3, transforming growth factor-beta 3; CTGF/CCN2, connective tissue growth factor/cellular communication network factor 2; RA, rheumatoid arthritis; PD, periodontal disease; LAP TGF-β1, latency-associated peptide of TGF-β1; CP, chronic periodontitis; PI, peri-implantitis; MMP13, matrix metalloproteinase-13; TIMP2, tissue inhibitor of metalloproteinases-2; TGFB3, transforming growth factor-beta 3 gene; OR, odds ratio; CBCT, cone-beam computed tomography; GAP, generalized aggressive periodontitis; OPG, osteoprotegerin; TNF-α, tumor necrosis factor alpha; RANKL, receptor activator of nuclear factor κB ligand; OC, osteocalcin; BSP, bone sialoprotein; COL I, collagen type I; Treg, regulatory T cells; Th17, helper 17 cells; IL, interleukin; IL-1β, interleukin-1 beta; IL-6, interleukin-6; IL-10, interleukin-10; IL-17, interleukin-17; IL-8, interleukin-8; CCL4, C-C motif chemokine ligand 4; CCL20, C-C motif chemokine ligand 20; ELISA, enzyme-linked immunosorbent assay; GCF, gingival crevicular fluid; PPD, probing pocket depth; IFN-γ, interferon gamma; CRP, C-reactive protein; HP, healthy patients; NSPT, non-surgical periodontal treatment; SRP, scaling and root planing; PI, plaque index; GI, gingival index; CAL, clinical attachment level; AgP, aggressive periodontitis; HtrA1, high temperature requirement A serine peptidase 1; HCMV, human cytomegalovirus; EBV, Epstein-Barr virus; CKD, chronic kidney disease; eGFR, estimated glomerular filtration rate; GTR, guided tissue regeneration; MMP-8, matrix metalloproteinase-8.

## Data Availability

All data generated or analyzed during this study are included in this published article.

## Abbreviations

The following abbreviations are used in this manuscript:

AgP	Aggressive Periodontitis
BSP	Bone Sialoprotein
CAL	Clinical Attachment Level
CBCT	Cone-Beam Computed Tomography
CCL4	C-C Motif Chemokine Ligand 4
CCL20	C-C Motif Chemokine Ligand 20
CKD	Chronic Kidney Disease
COL I	Collagen Type I
CP	Chronic Periodontitis
CRP	C-Reactive Protein
CTGF/CCN2	Connective Tissue Growth Factor/Cellular Communication Network Factor 2
EBV	Epstein-Barr Virus
eGFR	Estimated Glomerular Filtration Rate
ELISA	Enzyme-Linked Immunosorbent Assay
FDR	False Discovery Rate
GAP	Generalized Aggressive Periodontitis
GCF	Gingival Crevicular Fluid
GI	Gingival Index
GTR	Guided Tissue Regeneration
HCMV	Human Cytomegalovirus
HP	Healthy Patients
HtrA1	High Temperature Requirement A Serine Peptidase 1
IHC	Immunohistochemistry
IFN-γ	Interferon Gamma
IL	Interleukin
IL-1β	Interleukin-1 Beta
IL-6	Interleukin-6
IL-8	Interleukin-8
IL-10	Interleukin-10
IL-17	Interleukin-17
LAP TGF-β1	Latency-Associated Peptide of TGF-β1
MAPK	Mitogen-Activated Protein Kinase
MMP	Matrix Metalloproteinase
MMP-8	Matrix Metalloproteinase-8
MMP13	Matrix Metalloproteinase-13
NSPT	Non-Surgical Periodontal Treatment
OC	Osteocalcin
OPG	Osteoprotegerin
OR	Odds Ratio
PD	Periodontal Disease
PEA	Proximity Extension Assay
PI	Plaque Index/Peri-implantitis
PPD	Probing Pocket Depth
PRISMA	Preferred Reporting Items for Systematic Reviews and Meta-Analyses
qRT-PCR	Quantitative Real-Time Polymerase Chain Reaction
RA	Rheumatoid Arthritis
RANKL	Receptor Activator of Nuclear Factor κB Ligand
RCT	Randomized Controlled Trial
SMAD	Mothers Against Decapentaplegic Homolog (signaling proteins)
SRP	Scaling and Root Planing
TGFB	Transforming Growth Factor Beta Gene
TGFB3	Transforming Growth Factor Beta 3 Gene
TGF-β	Transforming Growth Factor Beta
TGF-β1	Transforming Growth Factor Beta 1
TGF-β3	Transforming Growth Factor Beta 3
Th17	T Helper 17 Cells
TIMP2	Tissue Inhibitor of Metalloproteinases-2
TNF-α	Tumor Necrosis Factor Alpha
Treg	Regulatory T Cells
